# Narrow-Linewidth 2 μm All-Fiber Laser Amplifier with a Highly Stable and Precisely Tunable Wavelength for Gas Molecule Absorption in Photonic Crystal Hollow-Core Fibers

**DOI:** 10.3390/molecules26175323

**Published:** 2021-09-01

**Authors:** Wenxi Pei, Hao Li, Yulong Cui, Zhiyue Zhou, Meng Wang, Zefeng Wang

**Affiliations:** 1 College of Advanced Interdisciplinary Studies, National University of Defense Technology, Changsha 410073, China; peiwenxi@nudt.edu.cn (W.P.); lihao18c@nudt.edu.cn (H.L.); cuiyulong@nudt.edu.cn (Y.C.); zhouzhiyue12@nudt.edu.cn (Z.Z.); wangmeng@nudt.edu.cn (M.W.); 2 Hunan Provincial Key Laboratory of High Energy Laser Technology, Changsha 410073, China; 3 State Key Laboratory of Pulsed Power Laser Technology, Changsha 410073, China

**Keywords:** 2 μm fiber lasers, master oscillator power-amplifier, high-power fiber lasers, narrow-linewidth, fiber gas lasers

## Abstract

In recent years, mid-infrared fiber lasers based on gas-filled photonic crystal hollow-core fibers (HCFs) have attracted enormous attention. They provide a potential method for the generation of high-power mid-infrared emissions, particularly beyond 4 μm. However, there are high requirements of the pump for wavelength stability, tunability, laser linewidth, etc., due to the narrow absorption linewidth of gases. Here, we present the use of a narrow-linewidth, high-power fiber laser with a highly stable and precisely tunable wavelength at 2 μm for gas absorption. It was a master oscillator power-amplifier (MOPA) structure, consisting of a narrow-linewidth fiber seed and two stages of Thulium-doped fiber amplifiers (TDFAs). The seed wavelength was very stable and was precisely tuned from 1971.4 to 1971.8 nm by temperature. Both stages of the amplifiers were forward-pumping, and a maximum output power of 24.8 W was obtained, with a slope efficiency of about 50.5%. The measured laser linewidth was much narrower than the gas absorption linewidth and the wavelength stability was validated by HBr gas absorption in HCFs. If the seed is replaced, this MOPA laser can provide a versatile pump source for mid-infrared fiber gas lasers.

## 1. Introduction

Fiber lasers operating at 2 μm are eye-safe and perform well in atmospheric transmittance and water absorption, so they have significant applications in laser radar, optical communication, surgery and other biomedical fields [1,2,3]. In the past two decades, they have been intensively studied [4,5,6,7,8].

Narrow-linewidth 2 μm fiber lasers are important pump sources for mid-infrared nonlinear frequency conversion [9,10,11] and gas lasers, as many gas molecules, such as CO_2_ and HBr have absorption lines in this waveband [12,13,14,15]. In the past decade, mid-infrared fiber gas lasers (FGLs) based on gas-filled hollow-core fibers (HCFs) have attracted enormous attention as they can generate laser emissions beyond 4 μm, which is currently very difficult for traditional rare-earth-doped fiber lasers [14,15,16]. In addition, because of their weaker nonlinearity and better heat dissipation capacity compared with solid-core fibers, FGLs are more likely to operate at higher power. In many experiments, high-power, narrow-linewidth fiber pump sources operating at a 2 μm waveband are required for gas absorption. Due to the narrow absorption linewidth of gas molecules, the typical value is currently several hundred megahertz. Although many high-power, narrow-linewidth fiber lasers operating at 2 μm have been reported, and their output power can reach a level of several hundred watts [17,18,19,20], wavelength stability and high-precision tunability are usually ingnored, which is cruicial for mid-infrared FGLs [15,16].

In this paper, we present the use of a high-power, narrow-linewidth 2 μm all-fiber amplifier with a highly stable and precisely tunable wavelength, based on a MOPA structure, which consisted of a narrow-linewidth fiber seed and two stages of TDFAs. The wavelength of the seed was very stable, and was precisely tuned from 1971.4 nm to 1971.8 nm by a thermal method. When the first-stage amplifier worked at ~1 W, the maximum output power of the second-stage amplifier was about 24.8 W, with a slope efficiency of 50.5%. The measured laser linewidth at the maximum output was much narrower than the absorption linewidth of HBr gas. The wavelength stability at maximum power was also validated by HBr gas absorption in HCFs. This work can provide a suitable high-power fiber pump source for mid-infrared FGLs based on HBr or CO_2_ molecules.

## 2. Experimental Setup

**Figure 1:**
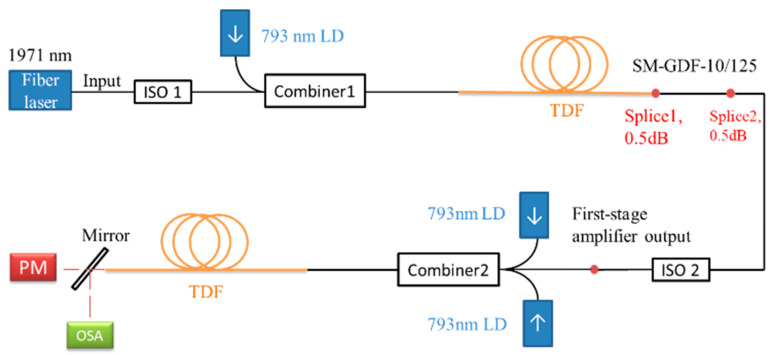
Experimental setup. ISO: isolator; PM: power meter; OSA: optical spectrum analyzer.

The experimental setup is shown in Figure 1. A customized 1971 nm single-frequency fiber laser (AdValue Photonics, Tucson, AZ, USA) worked as the seed, which was based on a fiber resonator formed by two fiber Bragg gratings. Under experimental conditions, it needs a sufficient warm-up to operate stably. An optical isolator (insert loss 0.78 dB and isolation 30 dB) was used to protect the seed and avoid damage by the backward laser. The seed laser and the 793 nm pump were combined by combiner1, then imported using 3-m-long thulium doped fibers (TDFs). The core diameter was ~10 μm and the absorption coefficient was 4.5 dB/m at 793 nm. Due to the mode field mismatch between the TDF and the input fiber of the optical isolator, a 0.5-m-long single-mode fiber (SM-GDF-10/125, Nufern) was introduced as the transition fiber to reduce the splicing loss. Compared with direct splicing, the total insertion loss was reduced by 0.5 dB, as shown in Figure 1. The residual pump laser was filtered out completely after the splice1 on the transition fiber. Another isolator (insert loss 0.5 dB and isolation 38.5 dB) was placed between the two amplifier stages to ensure the stability and safety of the first-stage amplifier. The output laser of the first-stage amplifier was used as the seed of the second-stage amplifier. The combiner2 had two pump input ends and both of them were used. The two LDs acted as the forward pump source to pump another 3 m long TDF, which had the same absorption properties as the TDF in the first-stage. The final output power and the spectrum of the MOPA were measured at the end of the TDF.

## 3. Experimental Results and Discussion

### 3.1. Seed Characteristics

**Figure 2:**
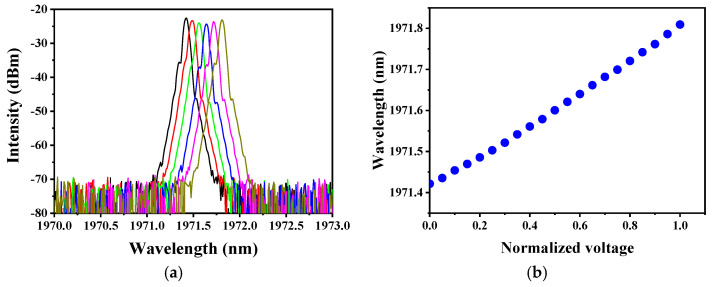
(**a**) The output spectrum of the seed under different controlling voltages. The corresponding central wavelengths from left to right are: 1971.42 nm, 1971.49 nm, 1971.56 nm, 1971.65 nm, 1971.72 nm, and 1971.81 nm, with normalized voltages of 0, 0.2, 0.4, 0.6, 0.8, 1, respectively; (**b**) The relationship between the central wavelength of the seed and the normalized voltage.

The seed used in our work was a commercially customized 1971 nm single-frequency fiber laser with a maximum output power of ~60 mW. The linewidth was narrower than 100 kHz and the beam quality was good (M^2^ < 1.1). Its central wavelength was adjusted by controlling the temperature with voltage. As the specific regulated voltage was unknown, the normalized voltage was used instead. Under different voltage conditions, the output spectrum of the seed was measured by an optical spectrum analyzer (OSA, AQ6375, Yokogawa, Tokyo, Japan, maximum resolution of 0.05 nm) and is shown in Figure 2a. The intensity difference in the figure is mainly due to measurement errors. Figure 2b presents the relationship between the central wavelength of the seed and the normalized voltage. By controlling the voltage, the wavelength of the output laser was tuned from 1971.4 nm to 1971.8 nm, and the minimum wavelength tuning step was due to the voltage controlling accuracy of the electric source.

### 3.2. The First-Stage Amplifier

We chose the forward pumping approach, which can provide a higher optical signal-to-noise ratio (SNR) than backward pumping. The seed was kept at the maximum output power, and the pump power increased gradually. The output power of the first-stage amplifier was measured at the output end of the isolator2 marked in Figure 2.

**Figure 3:**
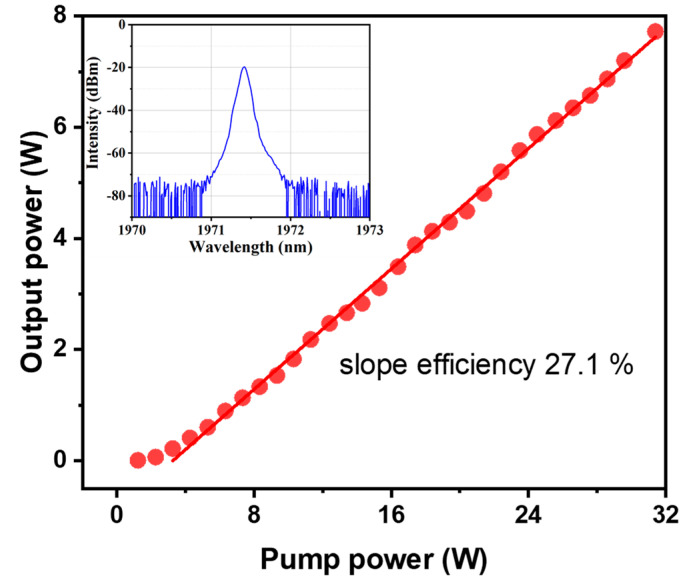
The output power of the first-stage amplifier and that of the 793 nm pump. Insert: the OSA-measured spectrum of the first-stage amplifier at maximum power.

As can be seen in Figure 3, the output power increased linearly with the pump power of the 793 nm LD. The maximum laser power was 7.6 W when the pump power was 31.4 W, with a slope efficiency of 27.1%. The insert in Figure 3 shows the output spectrum at this power, which maintained a high quality with no amplified spontaneous emissions (ASE). Besides, the output spectrum resembled the input spectrum and the central wavelength was 1971.42 nm. Due to the resolution of the optical spectrum analyser (OSA) the minimum resolution was 0.05 nm), we could not obtain the exact value of the linewidth.

### 3.3. The Second-Stage Amplifier

**Figure 4:**
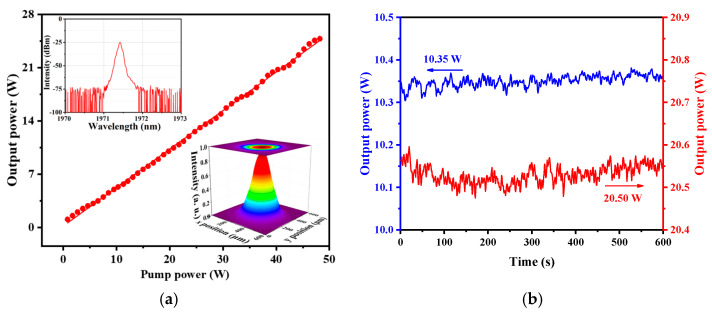
(**a**) The output power of the second-stage amplifier. The inserts in the upper left corner and bottom right corner are the output spectrum and the near-field pattern with the maximum output power; (**b**) The stability of the output power in 600 s.

The first-stage amplifier was used as the seed for the second-stage amplifier. The input power was controlled at ~1 W, which was beneficial for the power amplification and maintained good spectral quality. The output power characteristics of the second-stage amplifier is shown in Figure 4a. Similarly, the output power increased in line with the increase of the 793 nm pump’s power. The maximum output power was about 24.8 W, with a slope efficiency of about 50.5%, which was much higher than that of the first-stage amplifier. The difference in output power and the slope efficiency occurred due to the different input signal light power. In the first-stage amplifier, the input signal power from the seed was only ~60 mW, making the amplifier far from the saturated working state. However, in the second-stage amplifier, the input signal power was increased to ~1 W while the TDFs and pump source kept the same characteristics, which greatly accelerated the stimulated radiation process and made the amplifier approach the saturated working state, resulting in a higher slope efficiency. The output spectrum of the second-stage amplifier at maximum power is shown in the upper left corner of Figure 4a. Obviously, it resembled the spectrum of the first-stage amplifier, and showed the same good quality. Furthermore, there was no ASE. The insert in the bottom right of Figure 4a is the near-field pattern with the maximum output power, which indicates that the MOPA operated at fundamental mode.

Furthermore, to better explore the performance of the MOPA, the stability of the output power was measured, as shown in Figure 4b. After the MOPA was thermally stabilized, we measured the power fluctuations within 600 s with an output power of near 10.35 W and 20.55 W. Here we define the fluctuation ratio as the ratio of the difference between the maximum or minimum power and the average power to the average power in 600 s. The ratios were 0.74% and 0.59% respectively, which indicated the good stability of the MOPA. Meanwhile, the system showed good thermal stability during the process due to the necessary heat dissipation measures. When the seed was tuned from 1971.4 nm to 1971.8 nm, the MOPA showed similar power characteristics, and the maximum output power was limited by the available pump power.

**Figure 5:**
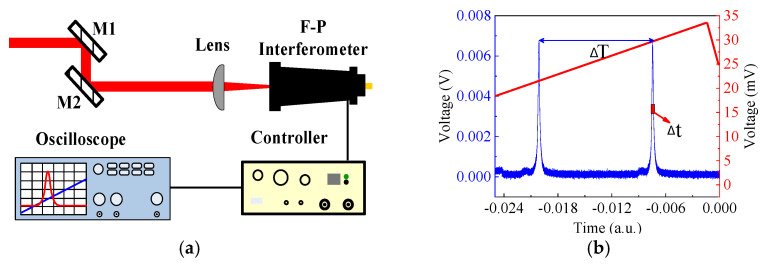
(**a**) Measurement setup for the laser linewidth using scanning Fabry-Perot interferometers; (**b**) Typical recorded line shapes by the oscilloscope.

Laser linewidth is a key parameter in gas absorption. In our experiment, we used a scanning Fabry-Perot (F-P) interferometer (SA200-18C, Thorlabs, Newton, NJ, USA, 1800~2600 nm, free spectral range (FSR) 1.5 GHz, resolution 7.5 MHz) to measure the linewidth of the MOPA, and the measurement setup is shown in Figure 5a. After being collimated and expended through the lens and mirrors, the output beam was coupled with the F-P interferometer. The controller amplified the signal detected by the interferometer and sent it to the oscilloscope. By measuring the full width at half maximum (FWHM, represented by Δ*t*) of the signal envelope and the time interval between the two adjacent signal envelopes (represented by Δ*T*), as marked in Figure 5b, the exact linewidth, Δ*ν*, of the output laser was calculated by Equation (1):

(1) $$\Delta \nu =FSR\frac{\Delta t}{\Delta T}$$

The measurement results are shown in Table 1, which were limited by the resolution of the F-P interferometer. Although we could not obtain the real linewidth, we did ensure that it was much narrower than the absorption spectrum width of most gas molecules used in mid-infrared fiber gas lasers, such as HBr and CO_2_, whose absorption linewidth is generally in the order of several hundred MHz at low pressure.

**Figure 6:**
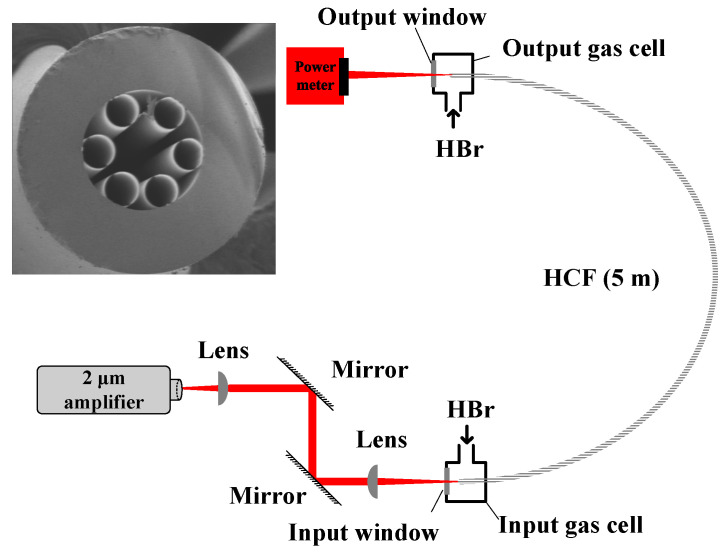
Measurement setup for the wavelength stability based on HBr gas absorption in HCFs. Insert: the scanning electron micrograph of the HCF’s cross-section.

**Figure 7:**
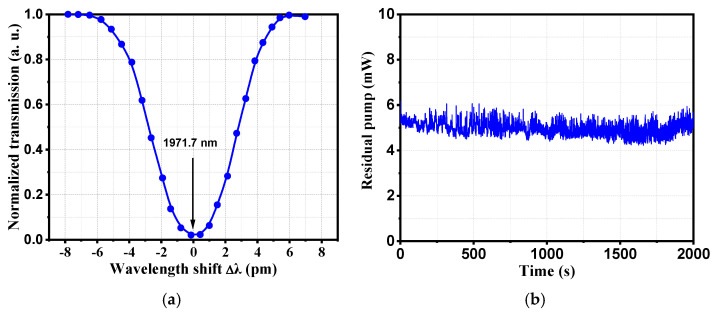
(**a**) Measured HBr gas absorption linewidth around 1971.7 nm at 10 mbar by fine-tuning of the seed wavelength; (**b**) the residual laser power after passing through the HBr-filled HCFs, with time at the centre of R(2) absorption line, implying wavelength stability.

For the application of gas absorption, there are high requirements for wavelength stability. To test the wavelength stability of the fiber amplifier in this work, gas absorption method based on HBr-filled HCFs was used because HBr gas has a strong absorption near 1971.7 nm, namely R(2) absorption line [16]. The experimental system is shown in Figure 6. A 5 m long HCF filled with 10 mbar HBr was sealed by two gas chambers. The insert in the figure is the scanning electron micrograph of the HCF’s cross-section. The output beam of the MOPA was coupled with the HCF by the lenses and mirrors. To avoid the generation of mid-infrared laser emission in the HCFs, during the experiments we attenuated the maximum output of the MOPA to about 120 mW, which was lower than the laser’s threshold. The linewidth of gas molecules was a Voigt profile, resulting from Doppler broadening, pressure broadening, and negligible natural broadening. During the measurement, we precisely tuned the seed wavelength across the R(2) absorption line and measured the residual power at the output window, so that the absorption linewidth of the HBr gas was estimated as shown in Figure 7a. The value was about 432 MHz. Subsequently, we kept the MOPA wavelength at the absorption centre near 1971.7 nm, and monitored the change of the residual laser power at the output. The results are shown in Figure 7b. They imply that the MOPA showed very good wavelength stability, because the residual power changed substantially if the laser wavelength shifted away from the absorption centre. Therefore, the MOPA met the need for gas absorption. It can be used as a high-power pump source for mid-infrared FGLs.

## 4. Conclusions

In this paper, we reported a high-power, narrow-linewidth 2 μm all-fiber MOPA with a highly stable and precisely tunable wavelength, which is the effective pump source for mid-infrared fiber gas lasers. Seeded by a thermal tunable fiber laser ranging from 1971.4 to 1971.8 nm, a maximum output power of 24.8 W was obtained after two-stage TDFAs, with a slope efficiency of about 50.5%, and the measured laser linewidth was only several MHz, which was much narrower than the gas absorption linewidth. The wavelength stability of the amplifier was validated by the absorption measurement of HBr gas in HCFs. By further optimizing the system parameters and increasing the pump power, the output power can be greatly improved. In addition, if we take place of the seed with different wavelengths, this MOPA laser can provide a versatile pump source for mid-infrared FGLs based on photonic crystal HCFs.

## Figures and Tables

**Table 1:** The evolution of the linewidth with the laser power.

Power (W)	Δ*T* (ms)	Δ*t* (μs)	Linewidth (MHz)
1	15.18	66	6.48
5	15.16	74	7.26
10	15.20	70	6.87
20	15.24	72	7.07

## Data Availability

The data presented in this study are available on request from the corresponding author. The data are not publicly available due to privacy restrictions.

## Sample Availability

Not available.
